# The efficacy and safety of acupuncture for Ramsay Hunt syndrome: A protocol for systematic review

**DOI:** 10.1097/MD.0000000000019582

**Published:** 2020-03-27

**Authors:** Ruijing Tan, Xingxin Yang, Xian Tang, Wenjie Ma, Lifen He

**Affiliations:** a Kunming Medical University, Kunming, Yunnan Province, China; b Yunnan University of TCM, Kunming, Yunnan Province, China; c Yunnan Center for Disease Control and Prevention, Kunming, Yunnan Province, China.

**Keywords:** acupuncture, Ramsay Hunt syndrome, systematic review protocol

## Abstract

**Background:**

Ramsay Hunt syndrome (RHS), also known as Hunt syndrome, is caused by varicella-zoster virus infection. The virus often invades the facial nerve geniculate ganglion to cause peripheral facial paralysis, accompanied by severe ear pain, auricular herpes zoster, tinnitus, deafness, vertigo, and other inner ear neurologic symptoms. The acupuncture has a long history as a traditional treatment of traditional Chinese medicine for the treatment of Hunt syndrome, with few adverse events and low cost. However, there are few evidences for the efficacy and safety of acupuncture for Hunt syndrome. Hence, we plan this systematic review and meta-analysis protocol to evaluate the efficacy and safety of acupuncture for Hunt syndrome.

**Methods:**

Four English databases will be searched from their inception to February 2019, including Cochrane Library, PubMed, Embase, OVID, and 4 Chinese databases, including Chinese Biomedical Literature Database [CBM], China National Knowledge Infrastructure [CNKI], CQVIP, and Wanfang. No restriction was imposed for language or publication period. Randomized controlled clinical trials (RCTs) compared any form of acupuncture with/without additional treatment against sham or no treatment or same additional treatment. Data will be extracted and evaluated by 2 reviewers independently. RevMan 5.3 software will be used for data analysis when a meta-analysis is allowed.

**Results:**

This systematic review and meta-analysis will provide an evidence of acupuncture for RHS.

**Conclusion:**

This study will determine whether acupuncture is an effective and safe intervention for RHS. PROSPERO registration number: CRD 42019118283.

## 1 Introduction

### 1.1 Description of the condition

The strict definition of the Ramsay Hunt syndrome is peripheral facial nerve palsy accompanied by an erythematous vesicular rash on the ear (zoster oticus) or in the mouth^[1]^ that may occasionally be associated with tinnitus, vertigo, deafness, severe otalgia, and inflammation of the pinna. The etiology of it is considered to be geniculate ganglionitis due to reactivation of varicella-zoster virus (VZV), analogous to herpes zoster occurring elsewhere in the body.^[2]^ The trigeminal nerve, the vestibulocochlear nerve, and the spinal ganglia C2–C4 are preferentially involved.^[3]^ Ramsay Hunt syndrome is the second most common cause of atraumatic peripheral facial paralysis.^[1]^ Before 1986, the frequency of zoster in patients with peripheral facial paralysis was estimated to be 4.5% to 8.9%.^[4]^ However, another retrospective review showed a frequency of identified Ramsay Hunt syndrome in patients presenting with unilateral facial palsy to be 12% based on the triad of facial paralysis, ear pain, and herpetic eruptions in any cranial dermatome.^[5]^ In addition, in a prospective study in Germany, researchers found a population-based incidence of 22.6 cases per 10,000 in 1992 to 1993.^[3]^ Although patients can recover after being treated with prolonged steroids^[6]^ or combination of steroid and antiviral agents,^[7]^ 15% to 30% of them still have complications including residual palsy and synkinesis.^[8]^

### 1.2 Description of the intervention

Acupuncture has been utilized in China for more than 3000 years as a unique technique in traditional Chinese medicine (TCM), and it has been practiced in treatment of substance addiction and nonsubstance addictions. Since 1980s, acupuncture is rapidly becoming part of mainstream medicine in the West. Patient visits to acupuncture clinics have dramatically increased.^[9]^ It is used to treat conditions, including pain, neurologic conditions, women's health, psychiatric disorders, cancer care, and functional bowel disorders.^[10]^ Particularly, acupuncture is widely used to alleviate various facial paralysis in clinical practice.

### 1.3 How the intervention might work

In TCM, it is believed that RHS is caused by abnormal function of qi activity that leads the exogenous pathogenic factors to invading Yangming, Shaoyang, and Taiyang Channels, resulting in blocking of facial meridians, disorder of qi and blood activity, and loss of nourishment of facial muscles and tendons.^[11]^ Acupuncture can recuperate qi activity, eliminate the exogenous pathogenic factors, and activate blood circulation to clear collaterals.^[11,12]^ Several researches have discussed the mechanism of acupuncture on facial paralysis. Zhao et al^[13]^ have concluded that acupuncture can adjust brain functional reorganization in motor cortex, and regulate the competitive activity between the hand area and face area in motor cortex to weaken the function in hand area and promote functional recovery in face area, which may be the mechanism underlying the acupuncture treating peripheral facial paralysis. Furthermore, Zhang et al^[14]^ have shown that in the acute stage of facial nerve injury, electroacupuncture can speed up the process of myelination of the neural nerve and promote the repair of the damaged facial nerve. Wang et al^[15]^ have found that the ShenDao superficial insertion by thick needle can improve the muscle contraction and relaxation and related energy metabolism of facial paralysis by significantly adjusting the expression of nerve and muscle-related genes, thereby restoring the normal state of the facial muscle group and nervous tissue of the body. Moreover, it is believed that acupuncture may work by improving the blood circulatory conditions to the malfunctioned nerve and removing the nervous conduction blockage via adjusting the meridian system.^[12]^

### 1.4 Why the intervention is important to perform this review

For treatment of RHS, antiviral medication in combination with corticosteroids is recommended.^[16]^ However, a Cochrane review^[7]^ has concluded that there is no evidence that antiviral agents have a beneficial effect on outcomes in Ramsay Hunt syndrome, despite their widespread use in this condition. On the contrary, corticosteroids are associated with a number of well-known adverse effects. So, acupuncture may be an option for treating RHS. The quality of clinical research has significantly improved, and a growing body of evidence shows the efficacy and effectiveness of acupuncture.^[9]^ Moreover, there are already several studies that have proved acupuncture to be a benefit treatment for facial palsy,^[17]^ including systematic reviews on acupuncture for bell palsy.^[18,19]^ However, there is no systematic review or meta-analysis on acupuncture for RHS.

### 1.5 Objective

This systematic review and meta-analysis will evaluate the efficacy and safety of acupuncture for Hunt syndrome.

## 2 Methods

This systematic review and meta-analysis protocol has been registered in PROSPERO (CRD 42019118283), and was performed according to Preferred Reporting Items for Systematic Reviews and Meta-analysis Protocol (PRISMA-P).

### 2.1 Eligibility criteria

#### 2.1.1 Types of studies

Randomized controlled clinical trials (RCTs) comparing any form of acupuncture with/without additional treatment against sham or no treatment or same additional treatment will be included. There is no restriction on language.

#### 2.1.2 Types of participants

Patients of any gender or age or race or nationality suffered from Ramsay Hunt syndrome will be included. Patients with severe complications associated with herpes zoster will be excluded.

#### 2.1.3 Types of interventions and controls

The experimental group should be treated with acupuncture (there is no limit on the needle materials, acupuncture points, stimulation techniques, stimulation methods, needle retention time, posture, stimulation frequency, and course of treatment) with or without antiviral medicine, nutritional nerve, and other basic treatment.

The control group should be treated with conventional antiviral therapy, nutritional nerve therapy, or other same basic treatment. However, trials in which the control group was given acupuncture therapy as well will be excluded.

#### 2.1.4 Types of outcomes

Primary outcome will include the number of participants with complete or partial recovery of facial neural function measured by total effective rate of patients. Effective should be identified as the facial paralysis is complete or partial recovered. Secondary outcome will include House-Brackmann grade, electroneuronography, pain intensity (measured by visual analog scale, numerical rating scale, McGill pain scale or other rating scales), adverse effects, and complications (including infection, needlesickness, needle breakage, and hematoma).

### 2.2 Data sources

Four English databases will be searched by 2 investigators (Xingxin Yang and Wenjie Ma) from their inception to February 2019, including Cochrane Library, PubMed, Embase, OVID, and 4 Chinese databases, including Chinese Biomedical Literature Database [CBM], China National Knowledge Infrastructure [CNKI], CQVIP, and Wanfang. There was no restriction for language or publication period. The list of references or citations found in secondary studies will be checked to identify possible eligible studies. When needed, the main authors of the studies will be contacted for more information.

### 2.3 Search strategy

The main terms “Ramsay Hunt Syndrome” and “acupuncture” indexed in MeSH, as well as the use of their synonyms and their crosses will be combined, for example, Search strategy in PubMed #1 Search “Herpes Zoster Oticus”[Mesh] #2 Search ((((((Herpes Zoster Oticus[Text Word]) OR Auricular Syndrome of Ramsay Hunt[Text Word]) OR Ramsay Hunt Syndrome[Text Word]) OR Geniculate Herpes Zoster[Text Word]) OR Herpes Zoster Auricularis[Text Word]) OR Herpes Zoster Cephalicus[Text Word]) OR Herpetic Geniculate Ganglionitis[Text Word] #3 #1 OR #2 #4 Search ((“Acupuncture”[Mesh])) OR “Acupuncture Therapy”[Mesh] #5 Search ((needling method[Text Word]) OR Acupuncture[Text Word]) OR electroacupuncture[Text Word] #6 #4 OR #5 #7 #3 AND #6.

### 2.4 Data collection and analysis

#### 2.4.1 Selection of studies

**Figure 1:**
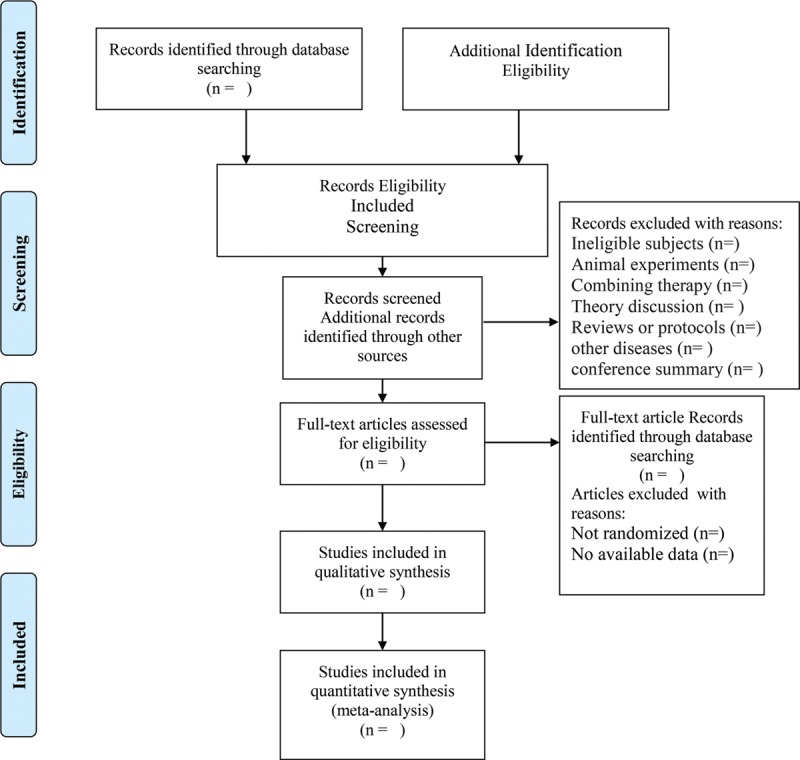
Flow chart of study identified.

Two reviewers will independently browse title and abstract of the literature primarily to exclude articles that do not meet the inclusion criteria, and then make a further judgment whether the articles that might meet the inclusion criteria could be included by going through the full text and then cross-check them. The disagreement will be determined by discussion or a third reviewer. The flow process of filtration is shown in a PRISMA flow chart (Fig. 1).

#### 2.4.2 Data extraction and management

After the screening is completed, 2 evaluators will independently extract data from the literature. The extracted content includes the study characteristics, details of the intervention and comparator used, outcome measures, and results. For articles published only in summary or articles whose important information is missing, we will seek complete information on the methods and results by contacting the authors. The disagreement will be determined by discussion or a third reviewer.

#### 2.4.3 Assessment of risk of bias and quality of studies included

Two reviewers will independently evaluate the methodological quality of included studies using the Cochrane Handbook's Risk of Bias Tool. The specific items are as follows: random sequence generation, allocation concealment, blinding, completeness of outcome data, selective outcome reporting, and other bias. The risk of bias for each item will be graded as “low risk of bias,” ‘unclear risk of bias,” or “high risk of bias.” Any disagreement will be resolved through discussion or the third reviewer.

#### 2.4.4 Measures of treatment effect

Dichotomous data will be expressed as risk ratio (RR) with 95% confidence intervals (95% CIs), and continuous data as mean difference (MD) or standard mean difference (SMD) with 95% CIs.

#### 2.4.5 Unit of analysis issues

According to the outcomes, effective rate of patients will be pool together. Secondary outcome will include House-Brackmann grade, electroneuronography, pain intensity, adverse effects, and complications (including infection, needlesickness, needle breakage, and hematoma).

#### 2.4.6 Dealing with missing data

For missing or incomplete data, we will contact the authors as much as possible. If we fail to obtain the missing data, we will detach the trails with missing data from the data synthesis. An intent-to-treat analysis will be performed if possible, and a sensitivity analysis will be performed to determine the impact of missing data.

#### 2.4.7 Heterogeneity assessment and data synthesis

The *I*-squared statistic and *P* values will be used to detect statistical heterogeneity among studies. Significant heterogeneity will be considered to be existed for *I*^2^ > 50%, and a value of *P* < .1 will be considered to be statistically significant. We will process the data of the study depend on whether they are suitable for meta-analysis. If the meta-analysis is not adaptive because of the heterogeneity of different participants, interventions, comparisons, outcomes, and so on, we will build a set of forms and summaries to conduct a qualitative description. If the meta-analysis is suitable for the data we will collect because of their good performance in homogeneity, we will apply the meta-analysis to conduct a quantitative analysis. Data will be analyzed using Review Manager (RevMan) software. If *I*^2^ < 50%, the fixed effect model will be selected; if *I*^2^ ≥50%, the random effect model will be selected. If considerable heterogeneity (*I*^2^ ≥ 75%) cannot be explained by the clinical and methodological diversity, the data will not be pooled.^[20]^ If the data dose not suit to quantitative analysis, the qualitative description will be conducted.

#### 2.4.8 Assessment of reporting bias

Reporting bias will be assessed using visual inspection of funnel plots for meta-analyses with 10 or more studies.

#### 2.4.9 Subgroup analysis

If there is a significant heterogeneity in the included studies, we will conduct subgroup analysis based on participants conditions, the intervention types, and comparison types (acupuncture vs sham acupuncture; acupuncture vs conventional intervention; acupuncture combined with conventional intervention vs conventional intervention).

#### 2.4.10 Sensitivity analysis

Sensitivity analysis will be used to identify whether the results are robust according to the heterogeneity and predefined criteria. The impact of methodological weakness, sample size, and missing data will be assessed. In addition, the data synthesis will be repeated after low-quality methodological trails are removed.

#### 2.4.11 Confidence in cumulative evidence

In this study, the quality of the evidence will be assessed by the Grading of Recommendations Assessment, Development and Evaluation (GRADE), which evaluate the quality of the evidence based on 5 factors, including study limitations, effect consistency, imprecision, indirectness, and publication bias. The assessment will be categorized into 4 levels: high, moderate, low, and very low quality.

#### 2.4.12 Ethics and dissemination

Ethical approval is not necessary, as this study is a systematic review. The findings of this review are disseminated through peer-review publication and conference presentations.

## 3 Discussion

RHS is a kind of acute facial nerve injury with peripheral facial paralysis. Facial paralysis is known as “deviated mouth” in TCM, which was attributed to “wind.” According to TCM, “Qi” refers to the vital substances comprising the human body and the physiological functions of viscera and bowels, channels and collaterals.^[21]^ Deficiency of “qi” allows the invasion of exogenous pathogenic wind.^[21]^ Acupuncture has been used to treat facial paralysis for several centuries as part of TCM. It has been shown that acupuncture treatment might have numerous beneficial effects for RHS in clinical. At present, there are already systematic reviews on acupuncture for some kinds of facial palsy but RHS. Insufficient evidence restricts the clinical use of acupuncture. So, it is meaningful to carry out this study. This review aimed to review systematically all randomized controlled trials comparing any form of acupuncture with/without additional treatment against sham or no treatment or same additional treatment to evaluate the efficacy and safety of acupuncture for Ramsay Hunt Syndrome. This systematic review and meta-analysis will provide convincing conclusion of whether acupuncture is effective and safe for RHS. We consider that the conclusion of this review will assist clinicians when treating RHS, and benefit patients with RHS. At the same time, it will provide clues to researchers to conduct further research in this subject area. The process of conducting this systematic review and meta-analysis will be divided into 4 parts: identification, study inclusion, data extraction, and data synthesis. If it is necessary to make changes or amendments, brief details of the changes made including date and corresponding reasons will be provided.

## Author contributions

**Conceptualization:** Ruijing Tan.

**Investigation:** Xingxin Yang, Wenjie Ma.

**Methodology:** Ruijing Tan, Xian Tang.

**Resources:** Lifen He.

**Software:** Ruijing Tan, Lifen He.

**Supervision:** Xingxin Yang, Xian Tang.

**Writing – original draft:** Ruijing Tan.

**Writing – review & editing:** Ruijing Tan, Lifen He.

Lifen He orcid: 0000-0002-7390-8736.
